# Progress in studies of epidermal stem cells and their application in skin tissue engineering

**DOI:** 10.1186/s13287-020-01796-3

**Published:** 2020-07-22

**Authors:** Ronghua Yang, Shuai Yang, Jingling Zhao, Ximin Hu, Xiaodong Chen, Jingru Wang, Julin Xie, Kun Xiong

**Affiliations:** 1 Department of Burn Surgery, The First People’s Hospital of Foshan, Foshan, 528000 Guangdong China; 2 Department of Neurosurgery, The First Affiliated Hospital of Sun Yat-Sen University, Guangzhou, 510080 Guangdong China; 3 Department of Burn Surgery, First Affiliated Hospital of Sun Yat-Sen University, Guangzhou, 510080 Guangdong China; 4 Clinical Medicine Eight-year Program, 02 Class, 17 Grade, Xiangya School of Medicine, Central South University, Changsha, 410013 Hunan China; 5 Department of Anatomy and Neurobiology, School of Basic Medical Science, Central South University, Morphological Sciences Building, 172 Tongzi Po Road, Changsha, 410013 Hunan China; 6 Hunan Key Laboratory of Ophthalmology, Changsha, 410008 Hunan China

**Keywords:** Epidermal stem cells, EPSC-dermal interaction, Skin tissue engineering, Skin regeneration

## Abstract

The epidermis, which is the outermost layer of mammalian skin, provides an essential barrier that is essential for maintenance of life. The epidermis is a stratified epithelium, which is maintained by the proliferation of epidermal stem cells (EPSCs) at the basal layer of the epidermis. As a unique cell population characterized by self-renewal and differentiation capabilities, EPSCs ensure the maintenance of adult skin homeostasis and participate in repair of the epidermis after injury. Recently, the utilization of EPSCs for wound healing and tissue regeneration has been attracting increased attention from researchers. In addition, the advances in tissue engineering have increased the interest in applying EPSCs in tissue-engineered scaffolds to further reconstitute injured tissues. In this review, we introduce research developments related to EPSCs, including methods recently used in the culture and enrichment of EPSCs, as well as advanced tools to study EPSCs. The function and mechanism of the EPSC-dermal units in the development and homeostasis of the skin are also summarized. Finally, the potential applications of EPSCs in skin tissue engineering are discussed.

## Introduction

Skin provides an interface between organisms and the outer environment, covering the entire surface area of the body. The epidermis is featured by a stratified structure and functions as a protective layer, including body temperature control, sensory perception, preservation of the hydration process, and protection against environmental assaults. Although the epidermis is capable of self-repairing and self-renewing, severe skin damages such as burn can impair the capacity for regeneration and may be life-threatening in severe conditions. As the skin is an immunocompetent organ, autologous skin grafts are the only feasible method to cover serious skin wounds permanently. Despite the development of tissue-saving autologous techniques of transplantation including Meek and mesh grafts, therapeutic strategies for extensive skin injury are seriously limited by the amount of skin available for grafting. Recently, the utilization of EPSCs in the skin regeneration has attracted the attention of researchers. EPSCs residing in the skin and its appendages, such as sweat glands and hair bulbs [1–3], are proliferation units that exhibit slow and limited division, can self-renew, and are responsible for generating the various lineages existing in the mature skin [4–6]. Currently, it is much easier to isolate and obtain well-characterized and specific EPSCs by using fluorescence-activated cell sorting (FACS) and a panel of biomarkers [7]. Moreover, the improvements of culture system for EPSCs, such as the combination of serum- and feeder-free media with small molecules, as well as the three-dimensional (3D) culture system, are important for EPSC expansion and maintenance of EPSC homeostasis in vitro. The use of combination of EPSCs with biomaterial scaffolds provides promising regenerative strategies for engineering skin, stem cell delivery, and regeneration of damaged skin tissues [8, 9]. In this review, we focus on the progress of techniques in EPSC research, introduce their interactions with different dermal cells, summarize their unappreciated important role as a niche, and highlight the function and mechanism of the EPSC-dermal units in the development and homeostasis of the skin. Finally, we introduce the development of biomaterial scaffolds and discuss the potential clinical applications of EPSC-based tissue-engineered skin in skin repair and regeneration.

## Culture and enrichment of EPSCs

### Methods for enrichment of EPSCs

The isolation and enrichment of EPSCs is challenging, mainly due to the insufficiency of biomarkers that distinguish EPSCs from other skin proliferative cells. Thus, it is essential to define methods and biomarkers to obtain well-characterized and specific EPSCs. As EPSCs have high integrin-β1 expression and collagen IV supports fast adhesion of EPSCs through integrin-β1, a relatively simple method for EPSC enrichment involves the incubation of cells on collagen IV for 20 min, and more differentiated cells with lower expression of integrin-β1 are rinsed away [10]. Fluorescence-activated cell sorting (FACS) is an efficient and widely used technique for EPSC selection [7]. Using FACS, EPSC enrichment can be achieved by sorting for high integrin β1 expression [10, 11] and the combination of high expression of integrin α6 with low transferrin (α6^high^/D71^low^) [12]. Other cell surface biomarkers, including delta-like 1 (DLL1) [13], CD46 [14], immunoglobulin-like domain 1 (LRIG1) [15], keratins19/5/14, CD49f^bri^/CD71^dim^ [16], Notch ligand, and leucine-rich repeats, have recently been identified and used in EPSC enrichment. Another FACS-based strategy for EPSC enrichment is sorting for cells that maintain the export of Hoechst dye [17].

In addition to isolating EPSCs from skin tissues, EPSCs can be generated from induced pluripotent stem cells (iPSCs) [18]. iPSCs, which share similar features to embryonic stem cells, can be obtained from adult somatic cells, including fibroblasts, keratinocytes, lymphocytes, and liver cells [19, 20] by exogenous addition of specific transcription factors, such as Sox2, Oct-3/4, c-Myc, and Klf4 using retroviral transduction. Recent studies have reported that EPSCs can be generated from human CD200+ and ITGA6+ iPSCs and can reconstitute hair follicle lineages and the interfollicular epidermis [21].

### Expansion of EPSCs in vitro

Cell culture is essential in stem cell-based tissue engineering and regenerative medicine. Several research groups have attempted to culture human epidermal cells, including EPSCs since the early 1960s; however, researchers failed to expand and subculture isolated EPSCs in vitro. Currently, the Rheinwald and Green method is the most widely used culture system for EPSCs, which allows massive expansion and progressive growth of colonies of cells by co-culturing EPSCs with mitotically inactivated feeder cells [22]. 3T3-J2 cells, which are a subclone of the original 3T3 line, are usually used as feeder cells, and mitomycin-C is used to treat feeder cells, which covalently cross-links double-stranded DNA and prevents the separation of DNA strands during replication [23]. In addition, the culture medium is improved by the addition of cholera toxin, adenine, triiodothyronine, epidermal growth factor, and adenine [23]. However, as the Rheinwald and Green culture method contains murine fibroblasts and fetal bovine serum, the transmission of microbes from animals is the potential risk, limiting its widespread application. To avoid this potential risk, chemically defined culture media without animal-derived ingredients have been developed and are commercially available [24]. These feeder- and serum-free media [25] enable clonal growth and serial culture of EPSCs, but it is still unclear whether they can maintain EPSCs for long periods ex vivo.

Recently, the components of the basement membrane, recombinant laminins LN-511 and LN-421, were used to replace murine 3T3 feeder cells [26], and this laminin culture system can be comparable to the murine cell co-culture systems in terms of colony-forming efficiency, gene expression, the capacity to form epidermis, and long-period maintenance of regenerated epidermis after transplantation, providing a new platform for EPSCs research and safer stem cell therapy. Furthermore, small chemical molecules, such as DMH-1 and A-83-01 (SMAD inhibitors) [27], RepSox (TGFβRI/ALK5 inhibitor) [28], DAPT (g-secretase inhibitor) [29], and Y-27632 (Rho-kinase inhibitor) [30, 31], are intracellular signaling inhibitors that can promote the expansion and proliferation of cultured EPSCs. The combination of serum- and feeder-free media with small molecules provides a new platform for EPSCs research and safer stem cell therapy.

Furthermore, new studies have attempted to utilize a three-dimensional (3D) culture system to mimic the stem cell niche that is important for EPSCs to maintain homeostasis. A 3D micronized (300–600 mm) amniotic membrane (mAM) was reported to retain the basement membrane structure and contain growth factors such as nerve growth factor (NGF), basic fibroblast growth factor (bFGF), hepatocyte growth factor (HGF), epidermal growth factor (EGF), and transforming growth factor-β1 (TGF-β1) [32]. EPSCs expanded quickly and maintained the characteristics of stem cells when cultured in the mAM, and the mAM with EPSCs gradually developed into a new epidermis when transplanted onto mice with full-thickness skin wounds [32].

## Tools to study EPSCs

### Genetic lineage tracing

Lineage tracing can identify the progeny of a single cell, proving the information of the number of all progeny of the founder cells and their differentiation status and location. As a crucial tool for studying the properties of stem cells, lineage tracing gives us information about how the stem cells behave in different organism.

The Cre-loxP system is usually used for genetic lineage tracing in mice [33, 34], which can genetic label stem cells in the tissue [35]. A transgenic mice expressing Cre recombinase fused to a mutated estrogen receptor expressed under the control of the epidermal basal layer-specific *keratin* 14 promoter was observed [36]. This mice line was crossed with a second mice line in which a reporter was flanked by a loxP-STOP-loxP (“floxed” STOP) sequence [37]. In mice that expressed both constructs, Cre recombines the loxP sites, excising the STOP sequence and activates LacZ expression genetically in the K14+ cells. The recombined and LacZ reporter is passed onto their progeny when these cells divide. By detecting β-galactosidase, the label can be observed [36]; however, β-galactosidase has its deficiencies because it cannot be tested in live cells by flow cytometry. The first fluorescent reporter mice are expressing enhanced GFP [38], and subsequently, other fluorophores are used, including tdTomato [39], enhanced yellow fluorescent protein, and multicolor confetti [40].

### Skin reconstitution assay

EPSCs are characterized by the capacity to self-renew and the ability to produce different differentiated cells in tissues, which are evaluated using the skin reconstitution assay [41]. Using this assay, the wound repair and reconstitution from disaggregated cell populations can be assessed [42]. In addition, compared to the lineage tracing, multiple parameters can be assessed and multi-lineage differentiation can be evaluated in a shorter time and using much fewer mouse. Several different methods for in vivo reconstitution of the epidermis have been investigated, such as seeding cells in a chamber between the epidermis and dermis [43] or seeding cells onto scaffolds [44]. The skin reconstitution assay not only allows clonal analysis, but also facilitates assessment of capacity of EPSCs to generate progeny and self-renew.

### Label retention

Stem cells including EPSCs are noted for their infrequent division (slow cycling) and relative quiescence. Since EPSCs are characterized by differential history of cell division, it could be distinguished from cells that divide frequently. As label-retaining cells (LRCs), EPSCs can retain radioactively-labeled nucleotides, such as thymidine analogs, tritiated thymidine, and 5-bromo-2′-deoxyuridine (BrdU) [45, 46]. The most commonly used DNA-label method is the BrdU pulse-chase method. The dividing cells can be marked and their division history can be followed by using this method [47]. As a pyrimidine analog of thymidine, BrdU can incorporate into DNA during DNA synthesis process. Once BrdU is injected onto mouse, cells that replicate during the pulse stage will be labeled. In the following “chase” stage, the frequently dividing cells lose most of their incorporated BrdU label after division, and EPSCs will keep the BrdU marker [46, 48]. However, the main disadvantage of this method is that some post-mitotic terminally differentiated cells will keep DNA markers efficiently and stay in the organs [49, 50]. Alternatively, to isolate and visualize LRCs, the pulse-chase technique and green fluorescent protein (GFP) tagged histone H2B (H2B-GFP) were used, which are expressed in specific tissues and tetracycline-dependent [51]. Currently, the tet-controllable transgenic mouse that expressed H2B-GFP has been used to mark and chase EPSCs [51]. This approach has now been used extensively as it can label quiescent cells completely, because of the earlier expressed H2B-GFP and relative longer pulse duration. Label-tracing experiments were performed not only in rodent models, but also in human cells [52, 53], showing that EPSCs are a state of slow cycle in vivo; however, when EPSCs are released from quiescence, they are capable of long-term proliferation and self-renewal [54].

### Clonogenic assays

Isolating cells from organs and culturing isolated cells in vitro is the earliest method used to identify EPSCs. Examination of the ability of clonal growth of individual cells can give us a quantitative read-out of EPSCs number and has been used to evaluate potential markers of EPSCs. Stratified epidermal cell sheets were formed by a subpopulation of proliferated cells, which form large colonies and at confluence [22]. The conditions for culturing EPSCs have been improved to enable the cultures to be used as autografts for the treatment of burn wounds, demonstrating that stem cells can survive in culture [55, 56]. The proliferative ability of primary epidermal cells are evaluated by culturing them at clonal density and then subcloned; three types of clones were identified [57], which prompted an attempt to identify the basis of their growth characteristics in vitro. One type of clone is termed “holoclones,” which are large circular colonies that generate more than 10^16^ cells when subcloned and have the capability of high proliferative potential and self-renew. The second type is termed “paraclones,” which are small and irregularly shaped clones. These cells do not have high proliferative potential generating new colonies and own the properties similar to transit amplifying cells. The third type of colony is called “meroclones,” which have proliferative ability and an appearance intermediate between paraclones and holoclones [57], demonstrating that not all basal cells in the epidermis have the same proliferative potential in vitro. In addition to these three identified types of clones, other detections for clonal growth have been employed, such as one in which the proportion of “abortive colonies” was scored. Abortive colonies obtain the features of transit amplifying cell founders; however, the clones that can self-renew actively obtain the features of stem cells [10, 58, 59]. When the proportion of putative stem cells is expanded, the proportion of cells that undergo terminal differentiation is unaltered [59].

## Interaction between EPSCs and the dermis

The heterogeneity and compartmentalization of EPSCs have been considered key features in epidermal homeostasis. As EPSCs existed in the basal layer of the epidermis are exposed to different types of dermal cells, including fibroblasts, adipocytes, muscle cells, neuronal cells, and melanophores, the previously unappreciated role of EPSCs as a niche for neighboring dermal cells has been highlighted recently [60]. The temporally and spatially specialized epidermal-dermal units play important roles in the development, function, homeostasis, repair, and regeneration of skin tissue.

### EPSC-fibroblast unit

**Fig. 1:**
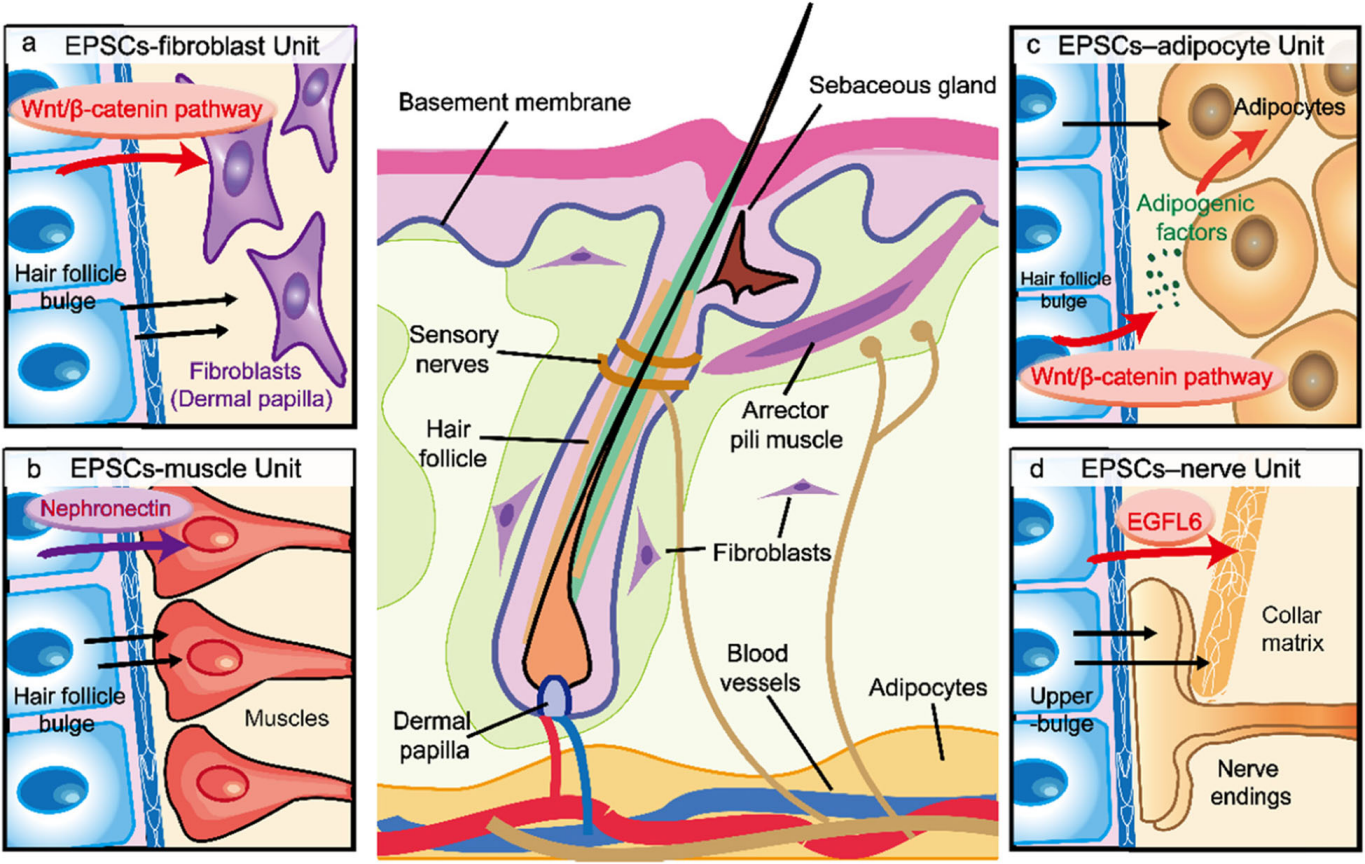
The interaction between EPSCs and the dermis. **a** The EPSC-fibroblast unit. **b** The EPSC-muscle unit. **c** The EPSC-adipocyte unit. **d** The EPSC-nerve unit. This image is based on a previously published image [60]

As well-characterized fibroblast populations, dermal papilla located at the base of each hair follicle and close to the basement membrane communicates with the neighboring epidermis and provides essential signals for hair follicle morphogenesis and regeneration [61–63]. As dermal papilla cells constantly attach to the hair germ epidermis, which contains EPSCs including shorter-lived transit amplifying cells and long-lived stem cells, thus, information from the epidermal layer are important for the activation and maintenance of dermal papilla (Fig. 1a). The transcriptional repressor Blimp1 and canonical Wnt signaling are key mediators of this interaction [63–65]. For example, upon induction of hair follicles during development, epidermal Wnt/β-catenin signaling induces Blimp1 expression in the dermal papilla fibroblasts through transforming growth factor (TGF) β signaling. In the dermal papilla, Blimp1 activates Wnt/β-catenin signaling and hair follicle stem cell-mediated hair follicle growth [66]. In addition, when the dermal papilla matures during hair follicle growth, the heterogeneity of mesenchymal signals including Wnt ligand gradients and bone morphogenetic protein (BMP) inhibitors creates distinct micro-niches along the epidermal-fibroblast interface in the dermal papilla [67]. Different basal cells in distinct epidermal structures can provide microenvironments to maintain and induce dermal fibroblast diversity; other epidermal appendages, including sweat glands, sebaceous glands, and nails also have different fibroblast populations under the epidermal layer, and further research is needed to clarify their characteristics and interactions [68].

### EPSC-muscle unit

A smooth muscle called the arrector pili muscle (APM) is involved in the association between the epidermis and the adjacent mesenchyme. APM is responsible for piloerection to trap warm air at the skin surface and thus plays an essential role in the thermoregulation. Piloerection also induces contraction of the sebaceous glands, enabling release of sebum onto the skin surface [69]. Hair follicle bulge is the permanent attachment site of the APM [70, 71] (Fig. 1b). The bulge containing EPSCs has been reported to construct a specific niche that facilitates the attachment of EPSCs to the hair follicle bulge and the development of APM [70]. During follicle development, bulge stem cells deposit a number of extracellular matrix (ECM) proteins onto the underlying basement membrane. Nephronectin is one of them, which is an ECM protein with five EGF-like repeats, a COOH-terminal MAM domain, and an RGD sequence [72]. As an important mediator of epidermal interactions with mesenchymal cells, nephronectin is specifically recognized by α8 integrin in dermal muscle precursors, promoting the differentiation of them and correct attachment to the bulge [72–74]. Detection of α8 integrin or nephronectin causes delocalization and reduced formation of the APM. In addition, research has shown that genes related to the formation of tendon/ligament are highly overrepresented in bulge EPSCs [70]. As the tendon or cartilage is aneural, avascular, and low-proliferative in the hair follicle bulge, extrinsic characteristics and tendon-like intrinsic in the bulge can explain the quiescent feature of bulge EPSCs.

### EPSC-adipocyte unit

Studies have shown that the oscillation in the dermal adipocyte layer and the growth cycle of hair follicle are synchronized [75]. In the anagen (growth) phase, the thickness of the adipocyte layer increases because of the hypertrophy of individual adipocytes and increased adipogenesis. In the catagen (regression) and telogen (resting) phase, hair follicles regress and the adipocyte layer becomes thinner [76]. Studies have shown that activation of the epidermal Wnt/β-catenin pathway correlates with the synchronized pattern of growth of hair follicle and expansion of the dermal adipocyte layer [77, 78] (Fig. 1c). Activation of epidermal Wnt pathway causes the secretion of adipogenic factors such as IGF signaling pathways and ligands of BMP [79]. Therefore, EPSCs produce transit amplify (TA) cells through Wnt signaling which are located at the bottom of the hair follicles, restricting the adipogenesis niche that reside at the lower part of the skin. However, activation of β-catenin pathway across the epidermal basal layer will lead to an extensive increase in adipocytes and failure in the development of hair follicle throughout the dermis [75]. Therefore, spatiotemporal activation of Wnt signaling in EPSCs provides an adipogenic niche in the lower part of the dermis, orchestrating the regeneration of two distinct cellular lineages during skin wound repair.

### EPSC-nerve unit

As the largest sensory organ in the human body, skin epidermis is innervated by different types of sensory nerves, which constitute a variety of mechanosensory end organ structures, such as Merkel cell-neurite complexes, hair follicle lanceolate complexes, and free nerve endings [80]. Lanceolate complexes, which consist of various non-cellular and cellular ingredients and form the collars of mechanoreceptor terminals, such as the terminal Schwann cell processes and parallel, longitudinally aligned low-threshold mechanoreceptor (LTMR) axonal endings [81], are exits in the upper bulge of the hair follicle, where Gli1-positive upper-bulge EPSCs reside [82–84] (Fig. 1d). As a sensory end organ, lanceolate complexes are very important in encoding several touch signals [85]. Studies have shown that EPSCs in the upper bulge of the follicle express a unique set of neurogenesis and ECM-related genes [86]. EPSCs deposit EGFL6, which is a kind of ECM protein, into the collar matrix. EGFL6 tightly ensheathes lanceolate complexes at the caudal side of the hair follicle. EGFL6 is essential for the touch responses, αv integrin enrichment of lanceolate complexes, and proper patterning. BDNF that derived from follicle EPSCs is essential for interactions of follicle-nerve, which is increased in the upper-bulge epidermis, and its deficiency destroys caudally polarized distribution of Aδ-LTMR lanceolate endings [84]. In addition, the EPSC-created epidermal tissue architecture also plays an important role in the formation, preservation, and function of the epidermal-nerve unit. The first bulge structure, which is established during follicle morphogenesis, is kept as an old bulge at the caudal side of the hair follicles, which provides a structurally stable niche for epidermal-nerve interaction and induces a lanceolate complex structure oriented toward the caudal side of the hair follicle [86].

## EPSCs in skin tissue engineering

Combining biomaterial scaffolds with stem cells is a potential strategy for engineering tissues, delivery of cells, and regeneration of native organs [8, 9]. Potential scaffold materials are classified as natural, such as collagens, hyaluronan, chitosan, silks, elastins, alginates, and fibrins [87, 88], and synthetic polymers including polyanhydrides, poly (lactic-co-glycolic) acid (PLGA), and polyethylene glycol (PEG) (Table 1). There are structural or functional classifications, such as whether they are injectable, hydrogels, surface modified capable of medicine delivery, by specific application. The most important characteristics critical to the success of biomaterial scaffolds are bioactivity, biodegradability, and biocompatibility [87]. These scaffolds suggest possibility for application of tissue engineering and could be used as substitutions for impaired organs. These scaffolds facilitate the differentiation and viability of stem cells seeded inside—based on both the incorporation of specific cues into the material and the intrinsic properties of the material.

**Table 1:** Scaffold materials in the tissue engineering

Biomaterials	Examples	Structure	Application
Natural biomaterials	Elastin	An elastic protein made up of water soluble tropoelastin	Cardiac stent coatings, soft tissue reconstruction, orthopedics
	Collagen	A fibrous triple-helical protein	Wound healing, skin grafts, muscle repair, nerve regeneration, anti-aging
		Collagen type I, a major subtype consists of two alpha 1 units and one alpha 2	
	Chitosan	A linear polysaccharide consisting of β-(1-4)-linked d-glucosamine and *N*-acetyl-d-glucosamine	Wound healing, orthopedics, cardiac repair, nerve regeneration, drug and gene delivery
Protein-based biomaterials	Fibrin	A fibrous non-globular protein produced by the cleavage of fibrinogen	Wound healing, cardiac repair, cell delivery
	Silk	Extracted from cocoon of silk worms. It contains fibrous protein fibroin and water soluble sericin protein	Muscle repair and regeneration, bone tissue engineering, cornea repair, drug delivery
Polysaccharide-based biomaterials	Alginate	An anionic polysaccharide consisting of homopolymeric blocks of (1-4)-linked β-d-mannuronate (M) and C-5 epimer α-l-guluronate (G) residues	Wound healing, drug delivery, soft tissue, engineering, cell delivery, in vitro stem cell maintenance
Synthetic polymer-based biomaterials	Poly-l-lactic acid (PLLA) and PLGA	Copolymers that consist of monomers of lactic acid and glycolic acid connected by ester bonds	Wound healing, cell delivery

### Natural biomaterials

It is advantageous to use natural biopolymers in biomedical applications, as these materials contain sites for cellular adhesion, they do not release cytotoxic degradation products, and the degradation rates of natural biopolymers can be manipulated [89]. Thus, many recent in vivo studies and the Food and Drug Administration (FDA) approval of new biomaterials for clinical use have utilized natural biopolymers as matrices for delivery of stem cells.

### Protein-based biomaterials

Protein-based scaffolds can provide appropriate structure in human tissues, which are applicable for tissue engineering involving the growth, differentiation, and transplantation of stem cells. As one of the major ECM-based proteins, collagen has been widely used for stem cell delivery in vivo. A recent study has reported that leucine-rich repeat-containing G-protein coupled receptor 6 (LGR6+) EPSCs are able to undergo proliferation, differentiation, and migration following seeding onto collagen-based scaffolds. These EPSC-containing collagen scaffolds are capable of repairing full-thickness wounds and hair regeneration [90]. Fibrin is a fibrinogen-derived protein, which has been used as a sealant in clinical researches and as a potential scaffold material for stem cell growth and delivery. Fibrin-based human skin substitute containing epidermal or dermal cells can result in good healing of massive full-thickness burn wounds. Another important ECM-based bio-macromolecule is elastin, which has also been used in a variety of applications of tissue engineering, including in the form of tropoelastin [91, 92]. In addition, scaffolds made of silk have slow degradation rates and desirable mechanical properties, making it attractive for tissue engineering applications. Scaffolds made from silk fibers can be fabricated into various structures, and silk can also be chemically modified. Stem cells, such as mesenchymal stem cells (MSCs), EPSCs, and adipose-derived stem cells (ASCs) combined with silk scaffolds can be successfully used in skin tissue engineering to enhance wound repair and regeneration [93, 94].

### Polysaccharide-based biomaterials

Polysaccharides, which are obtained from animal or plant sources and consist of sugar monomers, serve as a potential scaffold material for stem cell transplantation. One of the specific properties of polysaccharides is that they can rapidly be formulated into a gel, allowing them to be directly injected into injury sites. The use of polysaccharide-based scaffolds for the culture, differentiation, and delivery of stem cells in tissue engineering has been increasingly emphasized recently. Agarose, which consists of a galactose-based backbone, has been used as a scaffold in combination with stem cells in a variety of applications. Agarose scaffolds are appropriate for stem cell differentiation, providing a universal platform for tissue engineering [95]. Alginate, another polysaccharide, is derived from the cell walls of brown algae and can form scaffolds by ionic cross-linking, allowing the encapsulation of cells. Alginate scaffolds have also been used in combination with stem cells, such as adipose-derived adult stem cells and adult neural progenitor cells for repairing cartilage and spinal cord injuries [96, 97]. A recent study has also demonstrated that the viability, proliferation, and lineage differentiation of EPSCs are enhanced when they are encapsulated in alginate scaffolds [98, 99], and alginate can be used as an ideal bioink ingredient in stem cell-laden 3D bioprinting [100].

### Synthetic polymer-based biomaterials

Although natural biomaterials have been widely used in stem cell-based tissue engineering, these natural materials have some disadvantages, including a high risk for pathogen contamination, high lot-to-lot variability, and difficult sterilization and purification. In addition, these natural scaffolds have limited mechanical properties and need to be improved for stem cell culture [101, 102]. Synthetic biomaterials provide an alternative to natural materials to be used in stem cell culture. The defined chemical composition of these materials offers many advantages including reproducibility, controllable mechanical properties, and degradation rate. Therefore, a variety of synthetic biomaterials has been used for applications of different tissue engineering [87].

Poly-l-lactic acid (PLLA) and PLGA are copolymers that consist of monomers of lactic acid and glycolic acid connected by ester bonds. As approved polymers, PLLA and PLGA have been used in tissue engineering applications because of their ability to modulate the degradation rate and the biocompatibility. Scaffolds made using PLGA or PLLA were used to deliver stem cells to wounds, which can promote wound healing by enhancing the differentiation of stem cells [103, 104]. PEG is another biodegradable synthetic polymer that has been used for regenerative applications due to its high molecular weight and the ability to resist protein absorption. In combination with stem cells, PEG scaffolds have been widely used for their suitability as replacements for skin, cartilage, bone, liver, vasculature, and nerve tissues.

### 3D bioprinting

**Fig. 2:**
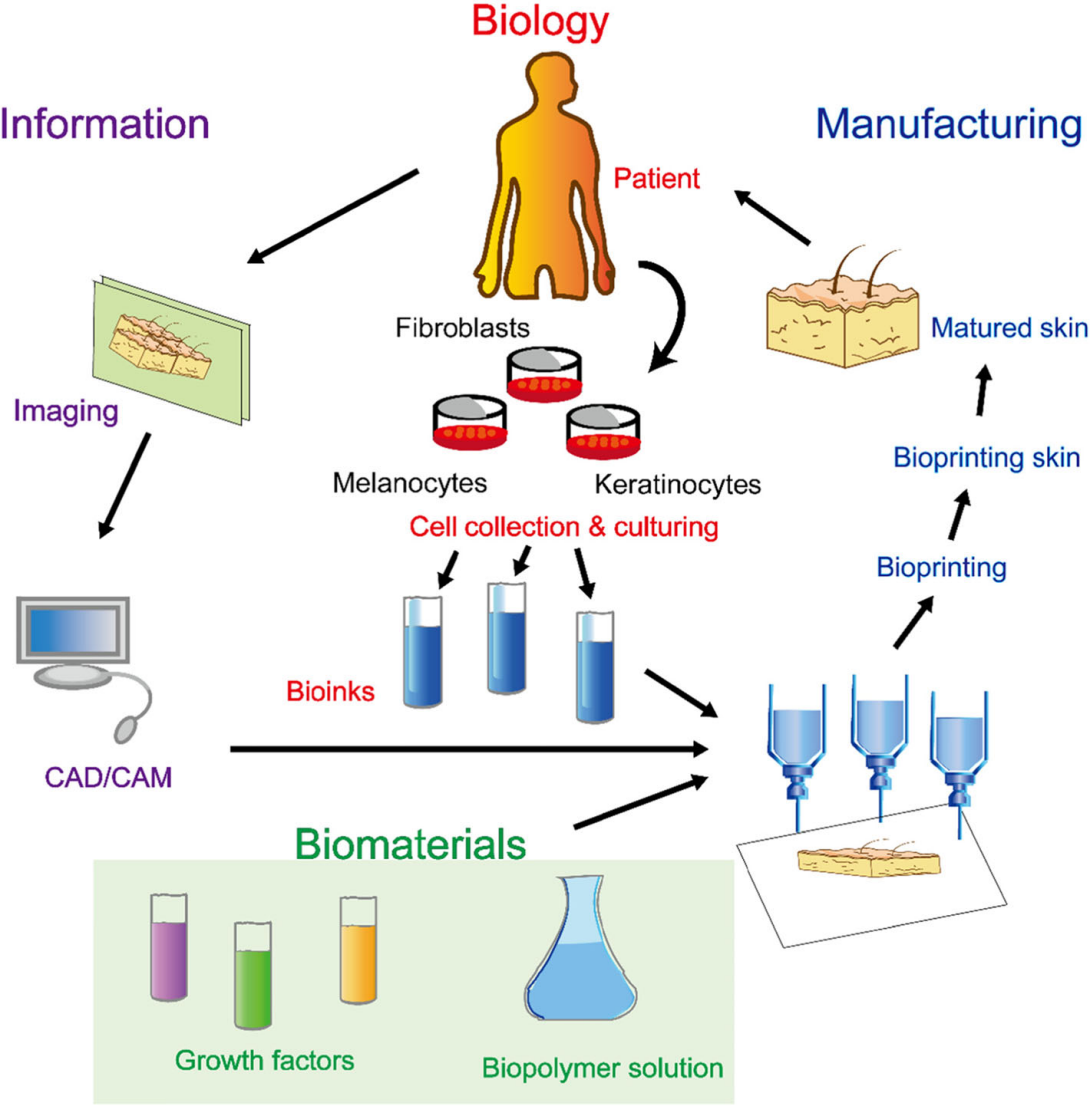
Steps in the fabrication of 3D bioprinted skin

3D printing technologies have been used to cells, supporting components, and biocompatible materials, giving a great promise for artificial organ and tissue printing (Fig. 2). Much of the 3D bioprinting research has been focused on reconstruction of human cartilage and bone tissues. More recently, there has been research into skin biofabrication using 3D bioprinting technology, which involves the use of deposition of stem cells, which was controlled by computer, into precise 3D geometrical patterns [105, 106]. Scaffold-based tissue engineering methods by cultivating and seeding stem cells in bioactive scaffolds have been widely used in reconstruction of skin. However, the deficiencies in these methods, such as lack of accurate delivery of cells, can be overcome by the use of stem cells combined with 3D bioprinting technology. Furthermore, the application of stem cells in the 3D printed constructs allow the introduction of various cell types with positional specificity [107]. The ability to “print” functional skin tissue successfully depends largely on the cell and biomaterial used. Similar to the bioink, the biomaterials should possess specific characteristics, including biocompatibility, biodegradability, bioinertness, strength, durability, and ductility [108]. These biomaterials are “printable,” which are determined by two characteristics: rheology and cross-linking abilities [109]. Therefore, the biomaterials used in 3D bioprinting are classified as natural polymers, including chitosan, collagen and fibrinogen, or synthetic polymers, including polycaprolactone and PLGA [110, 111]. In addition, cell selection is the second key component, and stem cells such as EPSCs have been identified as an ideal alternative as they have the ability of self-renewal and multiple differentiation potential [112]. Currently, the commonly used 3D bioprinting methods range from inkjet bioprinting, laser-assisted bioprinting, and extrusion bioprinting to much more advanced technologies including scaffold-free spheroid-based bioprinting [113]. For example, using the extrusion bioprinting method, cell-laden 3D-ECM mimics were bioprinted in vitro by using sodium alginate hydrogels and composite gelatin, which allowed for the EPSCs differentiation into sweat glands, and application of this bioprinted construct into skin wounds induces the reconstruction of sweat glands [114]. In addition, full-thickness skin equivalents have also been printed using the 3D inkjet bioprinting method combined with epidermal and dermal cells, with clinical and translational implications [115].

It is tangible to the application of 3D bioprinting in the wound repair and skin regeneration. The potential applications in dermatology are bioprinting skin models to test new treatments for diseases including atopic eczema [110], psoriasis, and vitiligo [116] and studying the pathophysiological mechanisms of skin wounds [117].

### FDA-approved products

Several tissue-engineered biological wound dressings have been approved by FDA. For example, Apligraf®, which was proved by FDA in 1998, is made from a bilayer structure, which contains a monolayer of keratinocytes at the outer layer and fibroblasts at the inner layer. As a living cell therapy, Apligraf® delivers the proteins produced by the cells and collagen and serves as a valuable therapeutic strategy for skin wounds, such as nonhealing venous leg ulcers (VLU) and diabetic foot ulcers (DFU) [118]. As a sister product of Apligraf®, GINTUIT® is a keratinocyte fibroblast-containing sheet, which is the first FDA-approved cell therapy product for wound healing in the oral soft tissue defects [119]. Similar to Apligraf, keratinocytes in GINTUIT® improve structural strength and fibroblasts provide the growth factors and the cytokine secretion effective on wound treatment through a bovine collagen matrix structure.

### Adipose-derived stem cells (ASCs) in skin tissue engineering

Studies have shown that ASCs not only can be used as a therapeutic method in personalized medicine to assess safety and efficacy of the breast reconstruction, but also have a potential usefulness in neurodegenerative conditions [120]. In addition, the fat graft enhanced with adipose-derived stem cells (FG-e-ASCs) has been widely used in repaired treatment of ulcers and breast tissue defects and breast augmentation [121]. Recently, engineered fat graft enhanced with adipose-derived stromal vascular fraction cells (EF-e-A) has been demonstrated as a reliable alternative to breast implant and used in breast reconstruction [122]. ASCs are recognized to promote wound healing through the inhibition of inflammation, promoting angiogenesis. Recent studies revealed that adipocyte-secreted exosomal microRNA (A-SE-MiR) also plays an important role in ASC-based therapies [123].

Autologous fat grafts have many clinical applications, such as facial rejuvenation, breast surgery, buttock augmentation, and Romberg syndrome. The stromal vascular cell fraction (SVF) of the adipose tissue provides a rich source of multipotent adipose tissue-derived stromal cells. The combined use of SVF/ASCs in fat grafting and platelet-rich plasma (PRP) is effective in the treatment of scars on the face, breast reconstruction, and chronic ulcers [124, 125]. PRP can release growth factors and hormones, representing an alternative strategy in wound repair and tissue regeneration. Clinically, PRP with or without Hyalomatrix PA (HPA) serves as new therapeutic strategies in the treatment of hidradenitis suppurativa, complex wound with bone exposure, and hair loss [124, 125]. In the treatment of hair loss, in addition to PRP, the use of stem cells, such as human follicle stem cells (HFSCs) and Human Intra- and Extra-Dermal Adipose Tissue-Derived Hair Follicle Stem Cells (HD-AFSCs), represents safe and effective therapies [126–128]. The activity of Wnt signaling in dermal papilla cells is important in enhancing growth of hair. In addition, signaling from platelet-derived growth factors and mesenchymal stem cells influences hair growth through cellular proliferation to prolong the anagen phase by FGF-7, stimulating development of hair follicle by β-catenin and cell growth by ERK activation, and suppressing apoptosis by Akt activation and Bcl-2 release [129, 130].

## Conclusions and perspectives

The currently used therapeutic strategies for skin repair are skin grafts and skin substitutes; however, there is still a lack of comprehensive treatment that emulates the complex regenerative process. As an ideal therapeutic product, the advanced bioengineered skin grafts containing EPSCs combined with biomaterials can efficiently support repair and regeneration of skin wounds. However, there are still many limitations on their experimental and clinical applications, for example, they are still expensive to manufacture, the skin appendices are usually not regenerated, and the experimental results of in vivo studies using rodents are only partially comparable to humans. Advanced stem cell research and tissue engineering approaches may provide solutions to these issues in the future. In addition to overcoming the difficulty of enrichment of renewable source of EPSCs in large quantities, major advances in both knowledge of the biology of EPSCs and the development of tissue engineering scaffolds will finally allow the widespread application of EPSC-based tissue-engineered skin for tissue repair and regeneration.

## Data Availability

Data sharing is not applicable to this article as no datasets were generated or analyzed during the current study.

## Abbreviations

EPSCs: Epidermal stem cells
FACS: Fluorescence-activated cell sorting
3D: Three-dimensional
DLL1: Delta-like 1
LRIG1: Immunoglobulin-like domain 1
iPSCs: Induced pluripotent stem cells
mAM: Micronized amniotic membrane
NGF: Nerve growth factor
bFGF: Basic fibroblast growth factor
HGF: Hepatocyte growth factor
EGF: Epidermal growth factor
TGF-β1: Transforming growth factor-β1
LRCs: Label-retaining cells
BrdU: 5-Bromo-2′-deoxyuridine
GFP: Green fluorescent protein
TGF: Transforming growth factor
BMP: Bone morphogenetic protein
APM: Arrector pili muscle
ECM: Extracellular matrix
TA: Transit amplify
LTMR: Low-threshold mechanoreceptor
PLGA: Poly (lactic-co-glycolic) acid
PEG: Polyethylene glycol
FDA: Food and Drug Administration
MSCs: Mesenchymal stem cells
PLLA: Poly-l-lactic acid
