# Correction to: Progress in studies of epidermal stem cells and their application in skin tissue engineering

**DOI:** 10.1186/s13287-022-02868-2

**Published:** 2022-05-05

**Authors:** Ronghua Yang, Shuai Yang, Jingling Zhao, Ximin Hu, Xiaodong Chen, Jingru Wang, Julin Xie, Kun Xiong

**Affiliations:** 1 Department of Burn Surgery, The First People’s Hospital of Foshan, Foshan, 528000 China; 2 Department of Neurosurgery, The First Affiliated Hospital of Sun Yat-Sen University, Guangzhou, 510080 Guangdong China; 3 Department of Burn Surgery, First Affiliated Hospital of Sun Yat-Sen University, Guangzhou, 510080 Guangdong China; 4 Clinical Medicine Eight-Year Program, 02 Class, 17 Grade, Xiangya School of Medicine, Central South University, Changsha, 410013 Hunan China; 5 Department of Anatomy and Neurobiology, School of Basic Medical Science, Central South University, Morphological Sciences Building, 172 Tongzi Po Road, Changsha, 410013 Hunan China; 6 Hunan Key Laboratory of Ophthalmology, Changsha, 410008 Hunan China

## Correction to: Stem Cell Res Ther (2020) 11:303 10.1186/s13287-020-01796-3

The authors wish to note the following in this correction article:

Figure 1 in the original article [1] was based on Fig. 3 in a previous article by Fujiwara et al. [2].

**Fig. 1:**
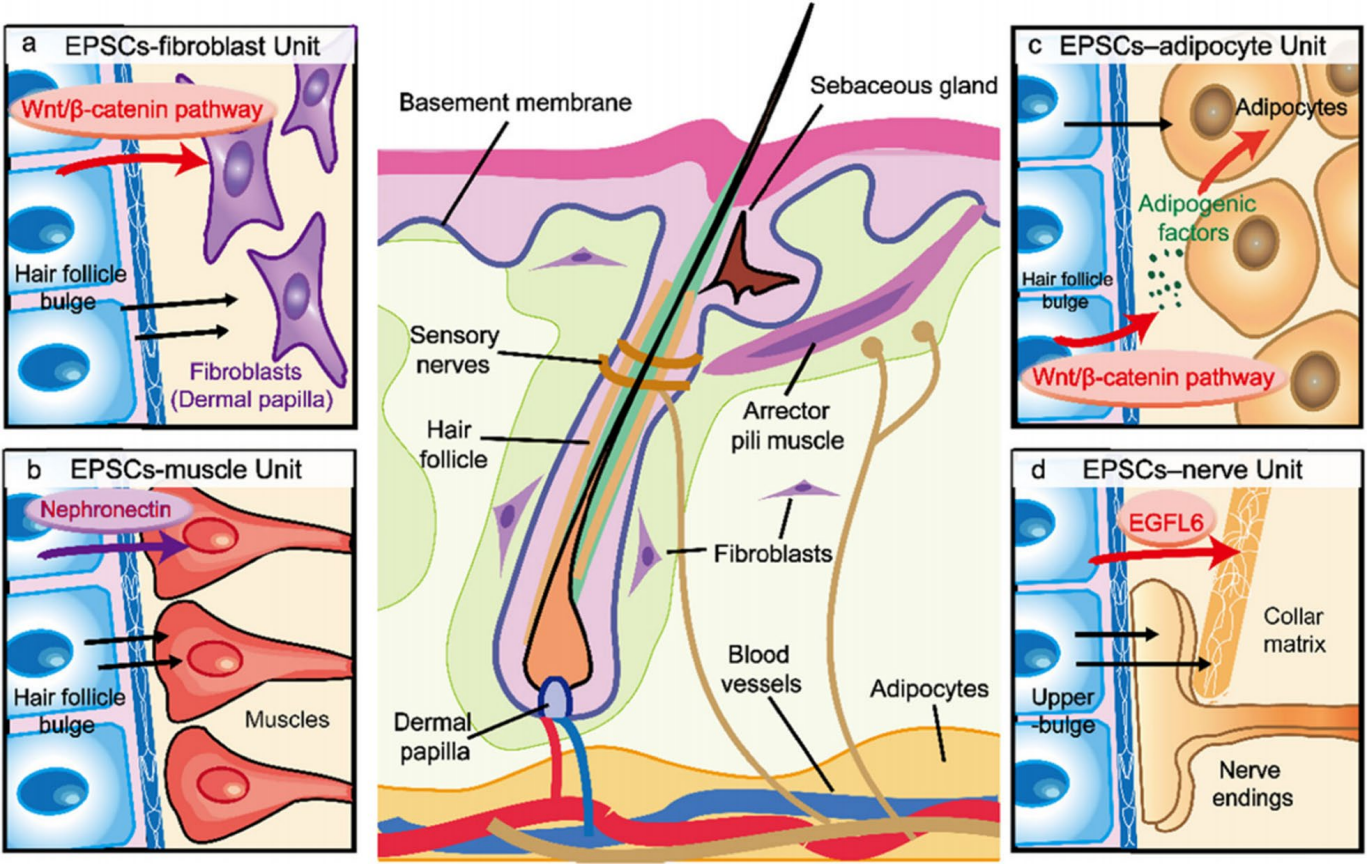
The interaction between EPSCs and the dermis. **a** The EPSC-fibroblast unit. **b** The EPSC-muscle unit. **c** The EPSC-adipocyte unit. **d** The EPSC-nerve unit. This image is based on a previously published image [2, 3]

## References

[1] Yang R, Yang S, Zhao J, Hu X, Chen X, Wang J (2020). Progress in studies of epidermal stem cells and their application in skin tissue engineering. Stem Cell Res Ther.

[2] Fujiwara H, Tsutsui K, Morita R (2018). Multi-tasking epidermal stem cells: beyond epidermal maintenance. Dev Growth Differ.

[3] Driskell RR, Watt FM (2015). Understanding fibroblast heterogeneity in the skin. Trends Cell Biol.

